# Global, regional, and national trends in 34 cancers through a novel burden–quality-of-care framework: a cross-sectional study

**DOI:** 10.1097/JS9.0000000000005067

**Published:** 2026-03-12

**Authors:** Lingkang Dong, Qiang Ma, Jiaxin Yang, Yihan Cheng, Wenqi Dong, Boya Zhang, Yuli Hu, Chao Du, Wen Lu, Dongzhen Yu, Haibo Shi

**Affiliations:** a Department of Otolaryngology Head & Neck Surgery, Shanghai Sixth People’s Hospital Affiliated to Shanghai Jiao Tong University School of Medicine, Shanghai, China; b Otolaryngology Institute of Shanghai Jiao Tong University, Shanghai, China; c Department of Nursing, The Sixth People’s Hospital Affiliated to Shanghai Jiao Tong University School of Medicine, Shanghai, China; d Department of Applied Social Sciences, Faculty of Health and Social Sciences, The Hong Kong Polytechnic University, Hong Kong, China; e Department of Neurosurgery, Beijing Tiantan Hospital, Capital Medical University, Beijing, China

**Keywords:** burden, cancer, cross-sectional study, global burden of disease, multidimensional framework, quality of care

## Abstract

**Background:**

Current unidimensional cancer evaluation systems limit precise cancer control. This study establishes a novel burden–quality framework that integrates disease burden and quality-of-care index (QCI) to better assess cancer management effectiveness globally.

**Methods:**

The QCI was derived by principal component analysis of six standardized indicators using the Global Burden of Disease data (1990–2021). A four-quadrant framework integrated QCI with disease burden to classify 34 cancers across 204 countries/regions. The sociodemographic index (SDI) and gender disparity ratios (GDR) were used to assess inequalities.

**Results:**

Substantial heterogeneity exists in the burden and QCI across 34 cancers. Highly fatal cancers such as TBL cancers (tracheal, bronchus, and lung cancers) and pancreatic cancers are persistently trapped in a “high burden-low quality” dilemma. In contrast, high-burden breast cancer and low-burden thyroid cancer demonstrate superior QCI, though the latter may carry overtreatment risks. Socioeconomic factors have a profound influence on intervention equity, with SDI exhibiting a strong positive correlation with QCI (*r* = 0.90, *P* < 0.001) but a significant negative correlation with GDR (*r* = −0.71, *P* < 0.001). Notably, the trend of QCI improvement and overall reduction in cancer burden is asynchronous: high-survival cancers and high-SDI regions showed slow QCI gains, middle-SDI regions exhibited substantial increases, while low-SDI areas remained persistently disadvantaged. Demographic disparities further exacerbated these inequities – older populations bore a disproportionate disease burden yet achieved the fastest QCI improvements, whereas adolescent groups maintained high baseline quality but stagnated in progress. Additionally, gender disparities were pervasive, with females receiving better-quality care for most cancers and across most regions.

**Conclusion:**

This multidimensional burden–quality framework shifts cancer evaluation from singular evaluation to precision intervention. It emphasizes: (1) optimizing resources for high-burden–low-quality cancers, (2) avoiding overtreatment in low-burden cancers, and (3) reducing inequities linked to socioeconomic disparities and vulnerable groups (especially elderly and male patients). This research provides a foundation for precise and equitable global cancer control.

## Introduction

Cancer prevention and control, as a core issue of global public health, confronts dual challenges of escalating disease burden and imbalanced resource allocation^[^1^]^. In 2022, global new cancer cases exceeded 20 million, with nearly 9.7 million deaths and showed epidemiological transition from high-income to low- and middle-income countries^[^2^]^. Despite significant international progress in early screening technologies^[^3^]^ and targeted therapy development^[^4^]^, substantial disparities persist across regions and populations in health care accessibility, quality-of-care equity, and prevention strategy effectiveness^[^5–7^]^.

The World Health Organization (WHO) defines quality of care (QoC) as the extent to which health care services improve desired health outcomes. It considers it a core indicator for Sustainable Development Goals (SDGs)^[^8^]^. However, current evaluation frameworks predominantly rely on unidimensional metrics, such as incidence/mortality rates, which may lead to deviations in health policy-making. This limited perspective fails to capture the dynamic relationship between QoC and cancer progression, constraining the development of precision prevention strategies.

To address these gaps, this study developed a quality-of-care index (QCI)^[^9^]^, establishing a novel burden–quality framework that integrates disease burden (disability-adjusted life years, DALYs) and care quality. Using principal component analysis (PCA) to reduce dimensionality, we synthesized six metrics (including incidence, prevalence, mortality, and DALYs) into a single index, thereby addressing the limitations of conventional indicators in capturing the “high burden-low quality” paradox. Based on Global Burden of Disease (GBD) data from 1990 to 2021, this research systematically evaluates spatiotemporal patterns across 34 cancer types and longitudinal panel data from 204 nations. We further incorporated the gender disparity ratio (GDR) and sociodemographic index (SDI) to characterize geographic and demographic heterogeneity in cancer-related health inequalities.

This study aims to: (1) establish a unified burden–quality framework for cancer burden and care quality; (2) identify priority “high burden-low quality” cancers, regions, and populations for intervention, as well as potential overdiagnosis and overtreatment in low-burden cancers; and (3) provide evidence-based strategies to optimize resource allocation and reduce inequalities across socioeconomic levels, age, and gender. The findings may help shift global cancer control from “homogeneous intervention” to “precision prevention,” offering crucial references for building a sustainable global cancer control system.

## Methods

### Data sources

This study utilized data from the GBD 2021, led by the Institute for Health Metrics and Evaluation at the University of Washington. The dataset encompasses epidemiological metrics for 371 diseases, 88 risk factors, and injuries across 204 countries and territories. This research collated data from multiple sources, including vital registration systems, epidemiological surveys, disease surveillance systems, cancer registries and open-access databases^[^10^]^. Rigorous harmonization and modeling techniques – such as meta-regression Bayesian regularized trimmed (MR-BRT), DisMod MR 2.1, and spatiotemporal Gaussian process regression (ST-GPR) – were applied to ensure robust and accurate estimates^[^10,11^]^. We systematically analyzed data from various countries and regions worldwide, stratifying them by gender and age group (<20 years, 20–54 years, and ≥ 55 years). All data were anonymized, and the University of Washington Institutional Review Board waived the requirement for informed consent. This cross-sectional study has been reported in line with the STROCSS guidelines^[^12^]^.

### Case definition

The study focused on 34 cancer types classified as Level 3 causes in the GBD hierarchy from 1990 to 2021, encompassing all malignant (ICD-10: C00–C97) and benign neoplasms. The ICD codes for related cancers have been listed in Supplemental Digital Content Table S1, available at: http://links.lww.com/JS9/H58.

SDI is a composite measure of income per capita, educational attainment, and fertility rate, and was used to stratify regions by socioeconomic development^[^10^]^. All 204 countries and territories were categorized into five SDI quintiles: high (> 0.810), high-middle (0.712–0.810), middle (0.619–0.712), low-middle (0.466–0.619), and low SDI (< 0.466).

HIGHLIGHTS

- This study proposes a novel four-quadrant burden–quality framework that integrates disease burden (DALYs) and a new quality-of-care index (QCI), providing a multidimensional assessment of global cancer management.
- Highly lethal cancers such as tracheal, bronchus, and lung cancers and pancreatic cancer remain in a persistent “high burden–low quality” trap, while thyroid cancer illustrates potential overtreatment risks despite excellent QCI performance.
- Socioeconomic disparities strongly influence outcomes: QCI correlates positively with SDI (*r* = 0.90) but inversely with gender equity (*r* = −0.71), suggesting that gender disparities in care quality are more pronounced in lower-SDI settings.
- Age- and region-specific heterogeneity was observed: middle-SDI regions achieved the fastest QCI gains, older adults showed the greatest improvements despite heavy burden, while adolescents stagnated in progress.
- Findings provide an evidence-based framework for precision intervention, guiding optimization of resource allocation, prevention of overtreatment, and targeted strategies to reduce inequities in vulnerable groups (elderly and male patients).

### QCI

QCI is a composite metric specifically designed to evaluate the quality of health care for a particular disease or condition. This index integrates multiple health indicators to reflect the performance of medical systems comprehensively^[^13–15^]^. The QCI calculation incorporates six core epidemiological parameters: mortality, incidence, prevalence, years of life lost (YLLs), years lived with disability (YLDs), and DALYs. To construct the QCI, these parameters were synthesized into four key age-standardized secondary indicators:

Mortality to incidence ratio (MIR) = $\frac{Death}{Incidence}$

DALYs to Prevalence (DPR) = $\frac{DALYs}{Prevalence}$

Prevalence to Incidence (PIR) = $\frac{Prevalence}{Incidence}$

YLLs to YLDs (YYR) = $\frac{YLLs}{YLDs}$

The mortality-to-incidence ratio (MIR) serves as an indicator of health care quality^[^13^]^. Under conditions of fixed incidence rates, elevated MIR values signify increased mortality, suggesting suboptimal care effectiveness. Larger values of the DALY-to-prevalence ratio (DPR) indicate that under the exact prevalence, the health loss caused by the disease is more severe. The prevalence-to-incidence ratio (PIR) functions as a measure for both prevention efforts and management efficacy^[^14,15^]^. Elevated PIR values may reflect either of two scenarios: (1) improved patient survival due to adequate care, or (2) inadequate prevention leading to case accumulation. A higher YLLs-to-YLDs ratio (YLR) indicates that premature death dominates the disease burden, potentially suggesting deficiencies in the health care system’s ability to prolong patient survival or a higher fatality rate of the disease itself^[^14^]^.

PCA is a statistical technique that simplifies data by reducing dimensionality and transforming correlated variables into a limited number of uncorrelated components, while preserving most of the original variance^[^16^]^. In this study, PCA was used to integrate these four secondary indicators into a single composite metric^[^15^]^. We retained the first principal component (PC1) as the underlying score for QCI. PC1 explained 75.40% of the variance at the country level and 82.07% at the cancer-type level, with bootstrap 95% confidence intervals (CIs) of 74.90–75.99 and 80.15–83.62, respectively. PCA diagnostics (including eigenvalues, variance explained, and PC1 loadings) are reported in Supplemental Digital Content Methods, available at: http://links.lww.com/JS9/H57.

PC1 exhibited positive loadings for MIR, DPR, and YYR, and a negative loading for PIR, indicating that higher PC1 values correspond to poorer care performance. We therefore rescaled PC1 to a 0–100 scale and reverse-standardized it so that higher QCI values consistently indicate better QoC. The age-standardized QCI was expressed as:

$$QCI=100-100^{*}\frac{{\text{PCA}}_{\text{score}}{\text{-PCA}}_{\text{min}}}{{\text{PCA}}_{\text{max}}{\text{-PCA}}_{\text{min}}}$$

By comprehensively analyzing data from 204 countries, 21 GBD regions, five SDI regions, and the global level, we constructed the QCI. To thoroughly assess population heterogeneity, we further calculated QCI values across different genders and age groups (<20 years, 20–54 years, and ≥55 years), aiming to reveal the distribution characteristics of health care accessibility and service quality among diverse populations. This approach provides a comprehensive and in-depth analytical perspective.

### GDR

We developed GDR to quantify sex-based differences in health care quality. This metric is derived from sex-stratified QCI data using the following formula:

$$GDR\;=\frac{{\text{QCI}}_{\text{female}}}{{\text{QCI}}_{\text{male}}}$$

The smallest differences in QoC between genders are represented by values close to 1. Values substantially greater than one indicate inequality favoring females, while values significantly less than one demonstrate disadvantage for females. In this study, we define 0.95 ≤ GDR ≤1.05 as the gender equivalence interval to enhance the reliability of the comparison.

### Four-quadrant analysis framework

To systematically evaluate the relationship between disease burden and QoC, we developed a four-quadrant analytical framework based on median thresholds of age-standardized DALYs rates (ASDR) and QCI. This approach categorizes cancer management and treatment into four distinct patterns: high burden–high quality, high burden–low quality, low burden–high quality, and low burden–low quality. This framework prioritizes cancers, regions, and populations characterized by a high burden and low quality that require urgent intervention, while simultaneously identifying optimally managed groups.

To assess the robustness of the median-based thresholds, we conducted systematic sensitivity analyses (Supplemental Digital Content Methods Tables S2–S4, available at: http://links.lww.com/JS9/H57), including (1) percentile-grid perturbation (p40–p60), (2) tertile-based cutoffs (p33/p67), and (3) mean ± 0.5 SD stress tests. Agreement across threshold schemes was quantified using stable% and Cohen’s κ. Under the 40–60% percentile perturbation, quadrant assignments remained highly stable: at the cancer-type level, stable% = 0.82–1.00 and κ = 0.76–1.00; at the regional level, stable% = 0.81–1.00 and κ = 0.74–1.00, supporting that the main conclusions were not driven by a single threshold choice.

### Statistical analysis

We comprehensively evaluated global trends in the burden of 34 cancers from 1990 to 2021, focusing on age-standardized rates (ASR) of DALYs and QCI. ASDR was reported per 100 000 population with 95% uncertainty intervals. ASRs were employed to account for varying age structures across regions and time periods, except for three predefined age groups (<20 years, 20–54 years, and ≥ 55 years) where crude rates were used since these analyses did not require cross-regional comparisons. The estimated annual percentage change (EAPC) and its 95% CI were calculated. EAPC was obtained by fitting a log-linear regression model using the formula^[^17,18^]^:

ln (ASR) = α + βx + ε

EAPC = 100 × (e^β^−1)

where x represents calendar year, α is the intercept, β reflects the annual change rate of ln(ASR), and ε is the error term. Trend significance was determined based on the 95% CI of EAPC: when the upper 95% CI limit was <0, it indicated a significant decreasing trend; when the lower 95% CI limit was >0, it indicated a significant increasing trend; and when the 95% CI included 0, the trend was considered to be stable. Detailed regression outputs and diagnostic checks for the EAPC models are provided in the Supplemental Digital Content Methods, available at: http://links.lww.com/JS9/H57.

Additionally, we used Pearson correlation coefficients to quantitatively evaluate the association between SDI and ASDR, QCI, and GDR across 21 GBD regions from 1990 to 2021, with visualization performed using the locally weighted scatterplot smoothing (LOESS) method^[^18^]^. All statistical analyses and graphical representations were conducted using the R statistical package version 4.2.3.

### External benchmarking of QCI

To evaluate the external convergent validity of QCI as a system-level quality signal, we benchmarked QCI against the Healthcare Access and Quality (HAQ) Index. Because HAQ estimates were available to 2019, we performed the comparison at two points (1990 and 2019) at the country/territory level (n = 204 per year; pooled n = 408). Associations were quantified using Pearson correlation (primary) and Spearman rank correlation (sensitivity).

In the pooled dataset, QCI was strongly correlated with HAQ (Pearson *r* = 0.925, 95% CI 0.909–0.938, *P* < 0.001; Spearman ρ = 0.940, *P* < 0.001) supporting the convergent validity of QCI.

## Results

### Burden and QoC for 34 cancers

#### Distribution and temporal trends in ASDR

**Figure 1.**
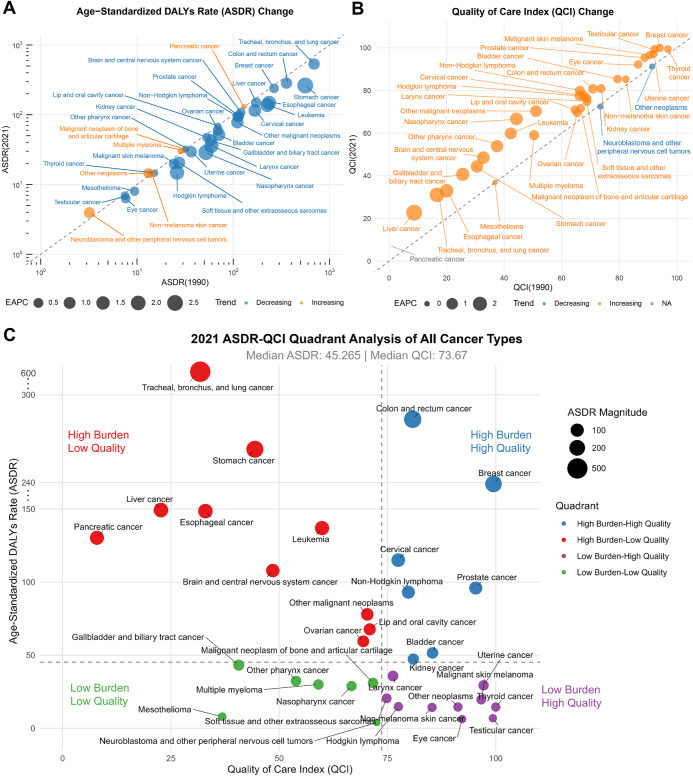
Comprehensive evaluation of 34 global cancer burden and quality of care (1990–2021). A. Temporal changes in ASDR for 34 cancer types (1990–2021). B. Temporal changes in QCI for 34 cancer types (1990–2021). The bubble plot compares ASDR or QCI values between 1990 (x-axis) and 2021 (y-axis), where the dashed diagonal line (y = x) denotes no change. Bubble sizes represent the absolute value of EAPC, where blue bubbles denote decreasing trends (EAPC < 0) and orange bubbles represent increasing trends (EAPC > 0). C. Quadrant analysis of 34 cancer burden and QCI (2021). The quadrant analysis evaluates the relationship between ASDR (y-axis) and QCI (x-axis), divided by median values (ASDR median = 45.265; QCI median = 73.67). Bubble size represents ASDR magnitude, with color-coded quadrants: red (high burden–low quality), blue (high burden–high quality), green (low burden–low quality), and purple (low burden–high quality). ASDR, age-standardized DALYs rate; EAPC, estimated annual percentage change; QCI, quality-of-care index.

In 2021, tracheal, bronchus, and lung (TBL) cancer (ASDR = 533), colon and rectum cancer (ASDR = 283.2), and stomach cancer (ASDR = 262.75) were the three leading contributors to cancer burden. In contrast, eight cancers, including testicular cancer (ASDR = 6.91), eye cancer (ASDR = 6.3), and mesothelioma (ASDR = 8.0), illustrate persistently low ASDR levels below 10/100 000. Trend analysis indicated that stomach cancer (EAPC = −2.54%), Hodgkin lymphoma (EAPC = −1.88%), and nasopharynx cancer (EAPC = −2.29%) demonstrated the most significant annual improvements. Non-melanoma skin cancer (EAPC = 0.24%) and neuroblastoma and other peripheral nervous tumors (EAPC = 0.74%) were among the two cancers showing significant upward trends (Fig. 1A; Supplemental Digital Content Table S2, available at: http://links.lww.com/JS9/H58).

#### Disparities and evolution in QoC

The QCI distribution exhibited a strong correlation with cancer prognosis (Fig. 1B; Supplemental Digital Content Table S2, available at: http://links.lww.com/JS9/H58). High-survival rate cancers, such as thyroid cancer (QCI = 100) and breast cancer (QCI = 99.48), demonstrated near-optimal care systems. In contrast, highly lethal cancers – including pancreatic cancer (QCI = 7.93), liver cancer (QCI = 22.7), and TBL cancer (QCI = 31.09) – persisted with low care quality. Notably, refractory cancers such as liver cancer (EAPC = 2.93%), lung cancer (EAPC = 2.23%), and oesophageal cancer (EAPC = 1.77%) showed the most rapid improvements in QCI. In contrast, Neuroblastoma and other peripheral nervous cell tumors, as well as mesothelioma, display no significant QCI gains over the past three decades (95% CI includes 0).

#### Association patterns between disease burden and QoC

Using median thresholds (ASDR median = 45.265; QCI median = 73.67), 34 cancers were categorized into four distinct burden–care quality patterns, revealing significant epidemiological and clinical management disparities (Fig. 1C; Supplemental Digital Content Table S2, available at: http://links.lww.com/JS9/H58).

High burden–high quality group (7/34 cancers): represented by breast cancer (ASDR = 239.03, QCI = 99.48), colon and rectum cancer (ASDR = 283.24, QCI = 73.67), and prostate cancer (ASDR = 95.94, QCI = 95.38). These cancers improvements in QCI excellent care quality despite substantial disease burdens.

High burden–low quality group (7/34 cancers): featured TBL cancer (ASDR = 533, QCI = 31.09), stomach cancer (ASDR = 262.75, QCI = 44.39), and pancreatic cancer (ASDR = 130.33, QCI = 7.93), demonstrating pronounced “high burden-low quality” disparities, particularly the notably poor care quality in pancreatic cancer.

Low burden–high quality (10/34 cancers): represented by thyroid cancer (ASDR = 14.57, QCI = 100), testicular cancer (ASDR = 6.91, QCI = 99.33), and uterine cancer (ASDR = 29.4, QCI = 97.19). These cancers had relatively low disease burden but high QoC.

Low burden–low quality group (10/34 cancers): comprised mesothelioma (ASDR = 8.0, QCI = 36.85), gallbladder and biliary tract cancer (ASDR = 43.2, QCI = 40.67), and other pharynx cancers (ASDR = 32.38, QCI = 53.89). Despite relatively low burdens, these cancers showed inadequate care quality, with neuroblastoma exhibiting increasing ASDR trends (EAPC = 0.74%) without quality improvement, suggesting potential clinical neglect.

#### Sex disparities in QoC

GDRs were analyzed for 29 non-sex-specific cancers, excluding five sex-specific cancers (see Supplemental Digital Content Table S2, available at: http://links.lww.com/JS9/H58). Overall, 72.4% (21/29) fell within the sex-equivalence range (GDR 0.95–1.05). Five cancers exhibited significant female advantage (GDR >1.05): pancreatic cancer (GDR = 1.58), other pharynx cancer (GDR = 1.23), brain and central nervous system cancer (GDR = 1.17), oesophageal cancer (GDR = 1.15), and nasopharynx cancer (GDR = 1.11). Conversely, gallbladder and biliary tract cancer (GDR = 0.84), mesothelioma (GDR = 0.85), and stomach cancer (GDR = 0.87) showed male advantage (GDR < 0.95).

### Global burden and QoC in all cancers

Between 1990 and 2021, the global cancer burden improved significantly (Table 1), with ASDR decreasing from 3969.21 to 2953.59 (EAPC = −1.04%). Concurrently, the QCI rose from 66.15 to 76.98 (EAPC = 0.51%). Sex-stratified analysis revealed that males had a 39.0% higher ASDR (3484.67) than females (2507.59), but they experienced faster QCI improvement (EAPC = 0.65% vs. 0.26% in females, respectively). In 2021, the global GDR was 1.16, reflecting significantly better overall care quality for females compared to males.

**Table 1:** Global trends in ASDR and QCI of all cancers by sex, age groups, and five SDI regions, 1990–2021.

	ASDR			QCI			GDR (2021)
Group	1990	2021	1990–2021 EAPC (95% CI)	1990	2021	1990–2021 EAPC (95% CI)	
Global	3969.21 (3792.05–4135.06)	2953.59 (2769.24–3154.03)	−1.04 (−1.07–1)	66.15	76.98	0.51 (0.48–0.54)	1.16
Sex							
Female	3285.33 (3089.49–3466.82)	2507.59 (2327.14–2686.76)	−0.98 (−1.03–0.94)	77.01	82.94	0.26 (0.24–0.28)	
Male	4793.62 (4492.53–5081.64)	3484.67 (3208.99–3814.81)	−1.09 (−1.13–1.06)	58.68	71.79	0.65 (0.62–0.69)	
Age group							
<20 years (crude rate)	631.63 (544.77–729.03)	354.47 (299.55–407.45)	−1.78 (−1.86–1.7)	86.15	83.44	−0.12 (−0.13–0.11)	1.31
20–54 years (crude rate)	2381.93 (2253.73–2496.15)	1938.72 (1814.2–2069.24)	−0.76 (−0.82–0.7)	70.68	69.82	−0.04 (−0.06–0.02)	1.13
55 + years (crude rate)	14 647.32 (13 955.76–15 273)	11 499.83 (10 652.32–12 323.63)	−0.87 (−0.91–0.83)	41.94	50.12	0.63 (0.57–0.7)	1.15
SDI region							
High SDI	4341.14 (4215–4430.83)	2920.6 (2751.48–3031.34)	−1.32 (−1.36–1.29)	80.76	87.18	0.23 (0.19–0.26)	1.05
High-middle SDI	4809.5 (4539–5055.08)	3388.27 (3078.37–3728.81)	−1.26 (−1.33–1.2)	61.9	78.54	0.86 (0.82–0.9)	1.21
Middle SDI	3778.75 (3482.1–4077.32)	2852.23 (2611.28–3158.67)	−1 (−1.04–0.96)	47.71	69.25	1.3 (1.27–1.34)	1.32
Low-middle SDI	2408.7 (2227.63–2562.93)	2376.66 (2202.92–2542.9)	−0.04 (−0.07–0.01)	51.05	62.01	0.68 (0.64–0.72)	1.34
Low SDI	2864.53 (2554.22–3184.28)	2487.39 (2164.52–2827.72)	−0.58 (−0.66–0.5)	37.32	49.74	1.08 (1.01–1.15)	1.41

ASDR, age‑standardized DALYs rate; EAPC, estimated annual percentage change; GDR, gender disparity ratio; QCI, quality-of-care index.

### Age-specific disparities in global burden and QoC in all cancers

Age-stratified analysis revealed a pronounced disease burden gradient (Table 1): the DALYs rate in adults aged 55 years and above (11 499.83) was 5.9 times higher than that in the 20–54 age group and 32.4 times higher than that in adolescents (<20 years). The adolescent group (<20 years) carried the lowest burden and showed the most rapid decline (EAPC = −1.78%), along with the highest but stable QCI (83.44; EAPC = −0.12%). In addition, although the ≥55 age group had the lowest QCI (50.12), it demonstrated the most tremendous improvement (EAPC = 0.63%). All age groups demonstrated a female advantage in care quality (GDR > 1.05), with the most pronounced sex disparity observed in adolescents (GDR = 1.31).

### Burden and QoC in all cancers by SDI region

From 1990 to 2021, high-SDI regions demonstrated the most remarkable achievements in prevention and control (Table 1), with the ASDR decreasing at an annual rate of 1.32%, resulting in a total reduction of 32.7% (from 4341.14 to 2920.60). In 2021, high-SDI regions achieved a QCI of 87.18, significantly surpassing other regions, though with relatively slow improvement (EAPC = 0.23%).

High-middle and middle-SDI regions shared notable catch-up trends. The ASDR decline in high-middle SDI regions (EAPC = −1.26%) approached that of high-SDI regions, while middle-SDI regions showed a QCI growth rate (EAPC = 1.30%) that was 5.6 times faster than high-SDI regions. Particularly noteworthy was the essentially stable ASDR in low- to middle-income SDI regions (EAPC = −0.04%), reflecting challenges in cancer control in these areas.

A transparent SDI gradient emerged in QoC, with QCI progressively increasing from 49.74 in low-SDI regions to 87.18 in high-SDI regions. All regions displayed GDR values greater than 1.05, indicating consistently better care quality for females. The most significant gender disparity was observed in low-SDI regions (GDR = 1.41), whereas high-SDI regions exhibited a relatively equitable distribution (GDR = 1.05), suggesting that gender inequities in health care access diminish with socioeconomic advancement.

### Burden and QoC in all cancers by GBD region

#### Global trends and geographic heterogeneity in ASDR

**Figure 2.**
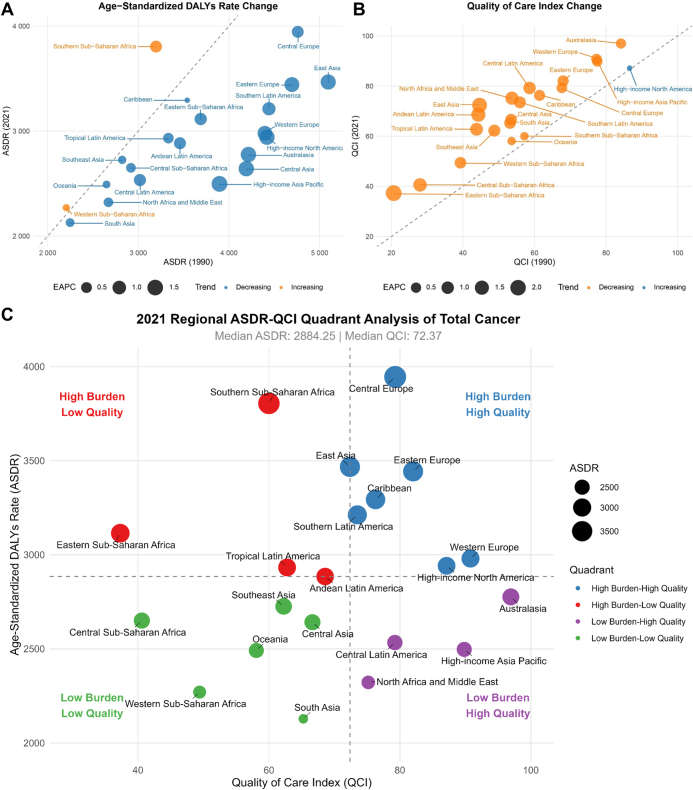
Comprehensive evaluation of all cancer burden and QCI across 21 GBD regions (1990–2021). A. Temporal changes in ASDR of all cancer burden across 21 GBD regions (1990–2021). B. Temporal changes in QCI of all cancer burden across 21 GBD regions (1990–2021). The bubble plot compares ASDR or QCI values between 1990 (x-axis) and 2021 (y-axis), where the dashed diagonal line (y = x) denotes no change. Bubble sizes represent the absolute value of EAPC, where blue bubbles denote decreasing trends (EAPC < 0), and orange bubbles represent increasing trends (EAPC > 0). C. Quadrant analysis of all cancer burden and QCI across 21 GBD regions (2021). The quadrant analysis evaluates the relationship between ASDR (y-axis) and QCI (x-axis), divided by median values (ASDR median = 2884.25; QCI median = 72.37). Bubble size represents ASDR magnitude, with color-coded quadrants: red (high burden–low quality), blue (high burden–high quality), green (low burden–low quality), and purple (low burden–high quality). ASDR, age-standardized DALYs rate; EAPC, estimated annual percentage change; GBD, global burden of disease; QCI, quality-of-care index.

Between 1990 and 2021, significant geographic disparities in global cancer burden were observed. The high-income Asia Pacific region (EAPC = −1.53%) and the high-income North America region (EAPC = −1.41%) demonstrated the most rapid declines in ASDR. Conversely, Southern Sub-Saharan Africa showed a significant increase in ASDR (EAPC = 0.66%), reaching 3804.26 in 2021. By 2021, Central Europe (3945.02), East Asia (3466.88), and Southern Sub-Saharan Africa (3804.26) remained high-burden regions, while Western Sub-Saharan Africa (2269.98) and South Asia (2128.19) maintained relatively lower burden levels (Fig. 2A; Supplemental Digital Content Table S3, available at: http://links.lww.com/JS9/H58).

#### Spatiotemporal evolution of QCI

Global QCI improvements displayed distinct regional patterns (Fig. 2B; Supplemental Digital Content Table S3, available at: http://links.lww.com/JS9/H58). East Asia (EAPC = 1.73%) and Eastern Sub-Saharan Africa (EAPC = 2.10%) showed the fastest growth rates, with East Asia’s QCI increasing from 44.66 to 72.37. High-income regions, such as Australasia (QCI = 96.95) and Western Europe (QCI = 90.78), maintained their global leadership but showed slower improvement rates. Notably, Sub-Saharan African regions remained at the lowest QCI levels, particularly Western Sub-Saharan Africa (49.41) and Eastern Sub-Saharan Africa (37.29).

#### Association patterns between disease burden and QoC

Using median thresholds (ASDR median = 2884.25, QCI median = 72.37), 21 regions were categorized into four burden–care quality patterns. High-income regions predominantly exhibited “low burden-high quality” characteristics (Fig. 2C; Supplemental Digital Content Table S3, available at: http://links.lww.com/JS9/H58).

High-burden–high-quality group (7/21 regions): represented by Central Europe (ASDR = 3945.02, QCI = 79.28) and Eastern Europe (ASDR = 3442.79, QCI = 82.02). These regions maintained relatively high care quality despite substantial cancer burdens.

High-burden–low-quality group (4/21 regions): featured Southern Sub-Saharan Africa (ASDR = 3804.26, QCI = 59.99) and Eastern Sub-Saharan Africa (ASDR = 3114.48, QCI = 37.29), demonstrating pronounced “high burden-low quality” disparities, particularly in the Eastern Sub-Saharan Africa.

Low-burden–high-quality group (4/21 regions): included high-income Asia Pacific (ASDR = 2496.98, QCI = 89.83) and Australasia (ASDR = 2776.10, QCI = 96.95), which represented global benchmarks with relatively low burdens and excellent care quality.

Low-burden–low-quality group (6/21 regions): comprised Central Sub-Saharan Africa (ASDR = 2650.01, QCI = 40.58), Western Sub-Saharan Africa (ASDR = 2269.98, QCI = 49.41), and Oceania (ASDR = 2491.25, QCI = 58.07). Despite relatively low burdens, these regions showed inadequate care quality, suggesting potential health care resource allocation inequities or health system inefficiencies.

#### Sex disparities in QoC

GDR analysis revealed that only five regions showed the tendency of falling within the gender-equivalence range (GDR 0.95–1.05). Sixteen regions demonstrated female advantage in care quality (GDR >1.05), while no regions showed male advantage. The most pronounced female advantage was observed in Sub-Saharan Africa (GDR = 1.40–1.72, see Supplemental Digital Content Table S3, available at: http://links.lww.com/JS9/H58).

### Burden and QoC in all cancers across 204 countries and territories

#### Trends and geographic heterogeneity in ASDR

**Figure 3.**
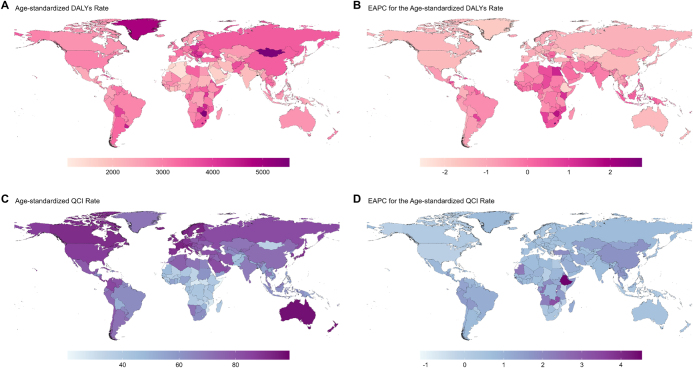
Global patterns of all cancer burden and care quality across 204 countries and regions. (A) ASDR in 2021; (B) EAPC of ASDR from 1990 to 2021; (C) QCI in 2021; (D) EAPC of QCI from 1990 to 2021. ASDR, age-standardized DALYs rate; EAPC, estimated annual percentage change; QCI, quality-of-care index.

Several nations, including Mongolia, Zimbabwe, Monaco, Lesotho, and Eswatini, maintained ASDRs exceeding 5000, indicating substantial cancer burdens. However, most countries demonstrated declining trends. South Korea (−2.60%), Kazakhstan (−2.49%), Singapore (−2.20%), Rwanda (−2.23%), and Ethiopia (EAPC = −2.04%) achieved the most significant decrease in ASDR, while Zimbabwe (3651.50 → 5397.50) and Southern Sub-Saharan Africa (3195.67 → 3804.26) experienced paradoxical ASDR increases (Fig. 3A–3B; Supplemental Digital Content Table S4, available at: http://links.lww.com/JS9/H58).

#### Global distribution and trends in QCI

Low-SDI countries generally demonstrated poor QCI performance, with Somalia (20.58), the Central African Republic (26.22), and Mozambique (26.59) representing global minima. Conversely, high-SDI territories like New Zealand (96.73), Australia (97.00), and Bermuda (98.95) approached theoretical maxima. Despite low baselines, several low-SDI nations, including Eritrea (15.31 → 28.83), Burundi (16.10 → 32.53), Zambia (18.59 → 36.29), and Equatorial Guinea (24.49 → 50.22), achieved rapid QCI improvements with EAPC exceeding 2%. In addition, Lesotho, Zimbabwe, Eswatini, and Kenya showed continued QCI deterioration (Fig. 3C–3D; Supplemental Digital Content Table S4, available at: http://links.lww.com/JS9/H58).

#### Global gender disparities QoC in all cancer

**Figure 4.**
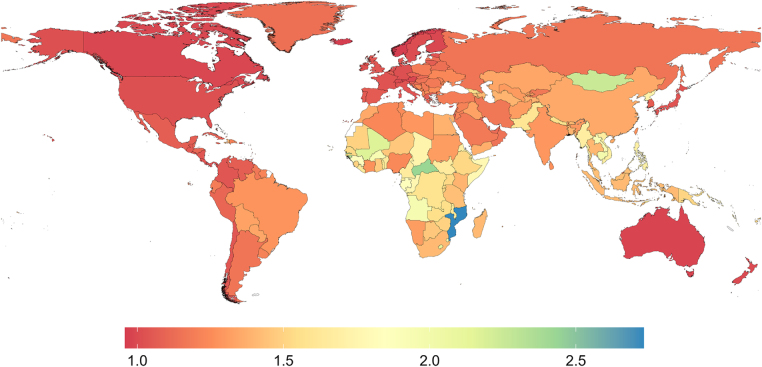
The gender disparity ratio (GDR) of all cancers across 204 countries and regions in 2021.

There are only 55 nations fell within the gender-equitable range (GDR 0.95–1.05). Several countries exhibited extreme gender imbalances, including Mozambique, Gambia, Central African Republic, Guinea-Bissau, Mongolia, Mali, Lesotho, Kiribati, Nauru, and Eswatini, where GDR values exceeded 2 (Fig. 4; Supplemental Digital Content Table S4, available at: http://links.lww.com/JS9/H58).

### Correlations between SDI and ASDR, QCI, and GDR

**Figure 5.**
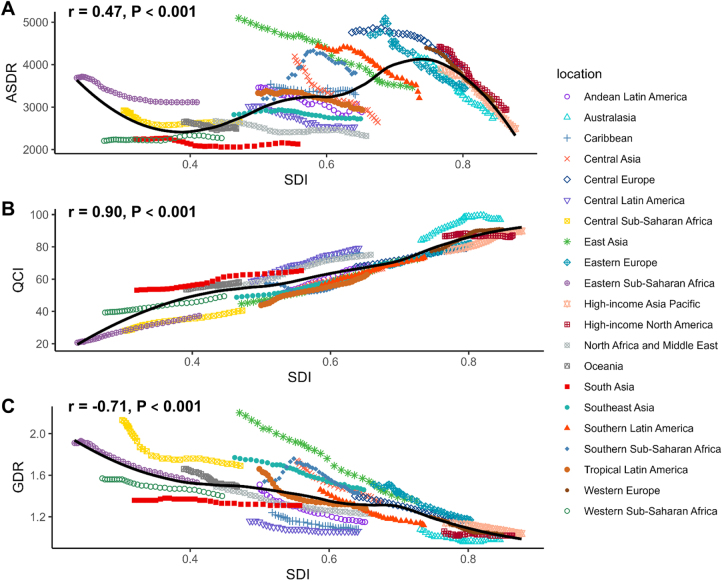
Correlation between SDI and ASDR, QCI, and GDR across 21 GBD regions from 1990 to 2021. (A) SDI and ASDR; (B) SDI and QCI; (C) SDI and GDR. Each point represents a region-year observation, with colors/shapes denoting regions. Black curves show LOESS trends. ASDR, age-standardized DALYs rate; EAPC, estimated annual percentage change; GBD, global burden of disease; GDR, gender disparity ratio; SDI, sociodemographic index; QCI, quality-of-care index.

For ASDR, although SDI showed a weakly positive correlation (*r* = 0.47, *P* < 0.001), LOESS analysis revealed a complex nonlinear relationship (Fig. 5A): negative correlations were observed when SDI<0.39 or SDI>0.74. In contrast, positive correlations appeared in the intermediate range (SDI 0.39–0.74). Regional stratification analysis further demonstrated that this overall correlation pattern was primarily attributable to geographic heterogeneity. Among 21 GBD regions, 19 showed significant negative correlations between SDI and ASDR (all *P* < 0.001). In contrast, Western Sub-Saharan Africa (*r* = 0.78, *P* < 0.001) and Southern Sub-Saharan Africa (*r* = 0.62, *P* < 0.001) exhibited positive associations (Supplemental Digital Content Figure S1, available at: http://links.lww.com/JS9/H59).

For other health metrics, SDI displayed a highly consistent positive correlation with QCI (*r* = 0.90, *P* < 0.001; Figure 5B), with the exception of high-income North America, representing the sole exception (Supplemental Digital Content Figure S2, available at: http://links.lww.com/JS9/H59). There is a significant negative correlation between SDI and GDR (*r* = −0.71, *P* < 0.001; Figure 5C), and this association remained robust across all 21 regions (Supplemental Digital Content Figure S3, available at: http://links.lww.com/JS9/H59).

## Discussion

This study establishes a burden–quality four-quadrant framework that jointly integrates cancer burden and QCI to characterize spatiotemporal patterns across 34 cancers, 21 regions, and 204 countries from 1990 to 2021. This dual-metric perspective reveals actionable “mismatch” scenarios – particularly high burden–low quality and low burden–low quality patterns – that can be obscured by unidimensional burden metrics. By integrating SDI and GDR, the framework helps identify populations and places where improvements in average performance may persistent inequities, thereby providing a policy-oriented tool for prioritizing precision interventions.

### Heterogeneous management and control paradigms across 34 cancer types

The “high burden–low quality” cluster, exemplified by TBL cancers and pancreatic cancer, remains a persistent global challenge. Part of this disadvantage is biologically driven (e.g., rapid progression, occult early symptoms, and limited efficacy of currently available therapies), which constrains achievable improvements even in well-resourced systems^[^19,21^]^. Meanwhile, the burden–quality gap may also be amplified by modifiable health-system constraints, including delayed diagnosis and unequal access to effective treatment, as health systems may prioritize cancers with effective screening and favorable prognoses^[^22^]^. These findings suggest that reducing the mismatch for such cancers likely requires both strengthened prevention/early detection where feasible and targeted improvements in treatment accessibility, alongside innovation for cancers with major biological limits.

In contrast, breast cancer illustrates a “high burden–high quality” model in which organized screening/early diagnosis and standardized multidisciplinary management can sustain high QCI despite substantial burden^[^23^]^. Thyroid cancer represents a typical “low burden–high quality” pattern; however, its rising incidence with declining mortality suggests that high-QCI settings should pair quality maintenance with value-based strategies to mitigate overdiagnosis and overtreatment^[^24,25^]^. For “low burden–low quality” cancers such as neuroblastoma and mesothelioma, the pattern may reflect neglect rather than intrinsic intractability: small case numbers can lead to low investment and limited clinical expertise^[^26,29^]^. Notably, quality metrics for high QCI cancers exhibit pronounced ceiling effects, where conventional improvement strategies yield diminishing returns. This impasse requires disruptive innovations, such as AI-assisted clinical decision-making^[^30^]^ and molecular pathology-guided personalized treatment protocols^[^31,35^]^, while balancing the harms associated with screening against the benefits of quality enhancement^[^36^]^.

Longitudinal trends from 1990 to 2021 further suggest that resource allocation should be differentiated: persistently poor-performing or worsening cancers may require strengthening of early detection and palliative care alongside access to effective treatment^[^37^]^; cancers with sustained improvement need quality-maintenance and equity-focused scale-up; and stagnated cancers warrant targeted research initiatives and implementation studies to identify barriers to progress^[^38^]^.

### Mismatch phenomenon and nonlinear relationship between QoC and cancer burden

Our four-quadrant framework highlights that QCI improvement and burden mitigation are not necessarily synchronized. High-survival cancers such as breast and thyroid cancers are consistent with the effectiveness of current control strategies, in which early detection and standardized treatment act synergistically to achieve near-optimal QCI values alongside sustained declines in ASDR^[^39,40^]^. Conversely, high-burden cancers like lung and gastric cancers show limited population-level burden improvement despite annual QCI gains exceeding 2%;, with ASDR remaining among the highest globally^[^41^]^. In addition, paradoxical ASDR increases in low-mortality tumors such as non-melanoma skin cancers highlight a critical challenge: gains in clinical care quality can be counteracted by rising exposures to environmental and behavioral risk factors^[^42,43^]^. Collectively, these patterns underscore the need to integrate public health prevention policies with efforts to improve health care quality.

### Global cancer control displays marked SDI gradient disparities

High-SDI regions achieved a 32.7% reduction in ASDR through systematic interventions^[^44^]^, yet exhibited 5.6-fold slower QCI growth than middle-SDI regions, consistent with diminishing returns in mature health systems. Low-SDI regions face dual challenges of stagnant ASDR and chronically depressed QCI, exposing systemic deficits in early diagnosis and treatment accessibility^[^45^]^. Middle-SDI regions exhibit compelling “catch-up effects,” exemplified by East Asia’s transition from “high burden-low quality” (QCI = 44.66) to “high burden-high quality” (QCI = 72.37, EAPC = 1.73%), demonstrating the potential for improving prevention and control levels through technology in underdeveloped regions. Paradoxically, middle-SDI regions face dual challenges: managing the rising cancer burden while bridging care quality gaps, and avoiding the development costs associated with repeating the experience of high-SDI regions^[^46^]^. This phenomenon warns against the “develop first, regulate later” approach, urging the institutionalization of primary cancer prevention measures simultaneously in the early stages of economic development^[^47^]^.

The strong correlation between SDI gradients and QCI (*r* = 0.90) is consistent with cumulative advantage effect in health care resource allocation. High-SDI regions establish virtuous cycles through the widespread adoption of early screening technologies and refinement of multidisciplinary diagnosis and treatment systems^[^48^]^, while low-SDI regions risk being trapped in a “high burden, low investment, low quality” vicious cycle. To address this governance challenge, we propose an SDI-stratified intervention strategy: (1) in low-SDI settings, prioritize foundational capacity building (screening/early diagnosis where cost-effective, pathology and staging, safe surgery, radiotherapy access, essential medicines, and palliative care); (2) in middle-SDI settings, scale up effective technologies while strengthening regulation, quality assurance, and financial protection to avoid inefficient “develop first, regulate later” trajectories; and (3) in high-SDI settings, emphasize value-based care, prevention of overtreatment, and innovation for hard-to-treat cancers in which biological constraints dominate.

### Spatial clustering patterns in cancer prevention and control

Global cancer epidemiology demonstrates marked geospatial clustering patterns in cancer burden and care quality. Australasia and high-income Asia Pacific dominate with “low burden-high quality” advantages, whereas Eastern and Southern Sub-Saharan Africa remain entrenched in “high burden-low quality” traps. WHO data show service availability gaps: over 90% of high-income countries provide essential cancer services, whereas 55% of low- and middle-income regions report that these interventions are unavailable^[^45^]^, with cost-related treatment discontinuation prevalent in low-income countries^[^49^]^.

### Demographic disparities in cancer burden and QoC

Elderly populations bear the greatest cancer burden yet receive the lowest care quality (only 60% of adolescent care levels), reflecting geriatric oncology that lags rapid population aging^[^50,51^]^. Adolescents show stagnant QCI improvements despite relatively higher baseline care quality, indicating early-control bottlenecks. Gender-specific analyses reveal overall female advantages (GDR = 1.16), particularly pronounced in pancreatic cancer and brain tumors, whereas males show advantages in gallbladder cancer and mesothelioma. Notably, these disparities intensify among adolescents (GDR = 1.31) and low-SDI populations (GDR = 1.41).

In-depth analysis reveals that socioeconomic factors profoundly influence gender equity in cancer management and treatment^[^52,53^]^. Based on the four-quadrant analysis, the most ideal gender parity (GDR ≈ 1) was observed in the high-burden–high-quality quadrant (e.g., breast cancer). At the same time, the most significant male disadvantage was found in the high-burden–low-quality quadrant. A significant negative correlation exists between SDI and GDR (*r* = −0.71): high-SDI regions achieve relative gender balance (GDR = 1.05), whereas low-SDI regions display severe imbalances (GDR = 1.41). These findings indicate that biologically based disparities are further amplified by socioeconomic factors, and that economic growth alone cannot entirely correct pre-existing gender imbalances. It underscores the urgent need to develop development-level-specific intervention strategies and implement precision prevention approaches tailored by age and gender.

### Forging a sustainable global ecosystem for cancer prevention and control

Based on the findings above, this study proposes four insights for building a sustainable global cancer control ecosystem: First, a conceptual shift from “burden-driven” to “quality-first” approaches is needed. Advances in cancer care quality do not align with the mitigation of disease burden. There is a need to establish a dynamic monitoring system that simultaneously focuses on burden control and quality improvement, avoiding the pursuit of short-term indicator gains at the expense of long-term health equity. Second, quadrant-specific strategies should be implemented across the 34 cancer types and 21 regions, with tiered response approaches for each quadrant types: high-burden–low-quality quadrant should prioritize cost-effective screening and expand access to basic diagnosis and treatment; low- burden–high-quality quadrant should focus on preventing over diagnosis and optimizing resource utilization; for cancers as well as regions with pronounced gender disparities, targeted male screening subsidy policies should be introduced to mitigate gender-based inequities in care quality. Third, a systematic approach to cancer control should be advanced by incorporating socioeconomic development, gender differences, and environmental policies into cancer management and treatment evaluation systems. Last but not least, a synchronized development-prevention mechanism should be established to strengthen comprehensive cancer control alongside socioeconomic development.

### Limitation

This study has several limitations. First, this study relies on GBD 2021 estimates; despite standardized modeling, cross-country variation in cancer registry coverage, coding practices, and reporting delays may introduce measurement error, particularly in low-SDI settings^[^54^]^. Second, associations between SDI and ASDR/QCI/GDR are not causal and may be affected by residual confounding because key determinants (e.g., risk-factor exposures and health-system inputs) were not jointly available for multivariable adjustment. Third, QCI cannot directly capture clinically granular domains such as surgical capacity, perioperative outcomes, timeliness of care, or palliative care coverage. Finally, quadrant assignment is based on relative cut-offs; although sensitivity analyses support robustness, the categories should be interpreted comparatively rather than as clinically defined thresholds.

## Conclusion

This study introduces a four-quadrant analytical framework to systematically characterize changes in the global cancer burden and QoC from 1990 to 2021. Key findings include: (1) heterogeneous burden–quality profiles across 34 cancer types, with highly fatal cancers such as TBL and pancreatic cancer persistently concentrated in a high-burden–low-quality pattern; (2) an asynchrony between QCI improvement and burden reduction, indicating that gains in care quality do not necessarily translate into proportional population-level burden mitigation; (3) pronounced population inequities, including quality gaps between older adults and adolescents and amplified sex disparities in resource-limited settings; and (4) a strong socioeconomic gradient, with diminishing returns in high-SDI regions, catch-up patterns in some middle-SDI regions, and persistently lower performance in low-SDI regions. Collectively, these findings underscore the need for quality-integrated surveillance and SDI- and quadrant-tailored interventions, prioritizing essential service capacity in low-SDI settings, quality governance and equitable scale-up in middle-SDI settings, and value-based care and innovation for hard-to-treat cancers in high-SDI settings

## Data Availability

All original data are accessible through the Global Burden of Disease Study 2021 at: https://www.healthdata.org/.
